# Circulating anti-angiogenic factors during hypertensive pregnancy and increased risk of respiratory distress syndrome in preterm neonates

**DOI:** 10.3109/14767058.2011.640368

**Published:** 2011-12-13

**Authors:** Alice Wang, Alexander M. Holston, Kai F. Yu, Jun Zhang, Mourad Toporsian, S. Ananth Karumanchi, Richard J. Levine

**Affiliations:** 1 Department of Pediatrics, Boston Medical Center, Boston University, Boston, Massachusetts; 2 Department of Pediatrics, Naval Medical Center Portsmouth, Portsmouth, Virginia; 3 Department of Mathematical Sciences, Tsinghua University, Beijing, China; 4 Shanghai Key Laboratory of Children's Environmental Health, Xinhua Hospital, Shanghai Jiaotong University School of Medicine, Shanghai, China; 5 Departments of Medicine; 6 Division of Pulmonary Critical Care and Sleep Medicine; 7 Department of Obstetrics and Gynecology, Beth Israel Deaconess Medical Center and Harvard Medical School, Boston, Massachusetts; 8 the Howard Hughes Medical Institute, Chevy Chase, Maryland; 9 Department of Health and Human Services, Eunice Kennedy Shriver National Institute of Child Health and Human Development, Division of Epidemiology, Statistics, and Prevention Research, Bethesda, Maryland; 10 Deceased

**Keywords:** Anti-angiogenic, soluble fms-like tyrosine kinase 1, sVEGF R1, sFlt1, placental growth factor, PlGF, soluble endoglin, sEng, respiratory distress syndrome, RDS, neonate, preterm, preeclampsia, gestational hypertension

## Abstract

*Objective:* To test the hypothesis that high circulating concen-trations of maternal anti-angiogenic factors are associated with increased risk of respiratory distress syndrome (RDS). *Study Design:* This is a nested case-control study of nulliparous women who delivered less than 37 weeks of gestation within the Calcium for Preeclampsia Prevention (CPEP) trial. The study included 116 women with preeclampsia or gestational hyperten-sion and 323 normotensive controls. Soluble fms-like tyrosine kinase 1 (sFlt1), placental growth factor (PlGF) and soluble endo-glin (sEng) in maternal serum were measured at 21–32 weeks of gestation. *Results:* Preterm infants born to hypertensive mothers were more likely to develop RDS (22.5% vs. 20.9%, *p* =0.03). After adjustment for gestational age at delivery, the odds ratio for the relationship between hypertension in pregnancy and RDS was 2.18 (95% CI 1.08–4.39). In hypertensive pregnancies women whose infants developed RDS had significantly higher circulating mean sFlt1 levels during midpregnancy (21–32 weeks of gestation) even after adjustment for gestational age at delivery (21,516 pg/mL vs. 7,000 pg/mL, *p* =0.01). *Conclusions:* Preterm preeclampsia and gestational hypertension, charac-terized by high circulating levels of sFlt1, are associated with a twofold increased risk of RDS in infants delivered before 37 weeks. Among women with these hypertensive pregnancies circulating sFlt1 concentrations during midpregnancy were substantially higher in women whose infants developed RDS.

## Introduction

Preeclampsia is a progressive disease characterized clinically by new onset hypertension and proteinuria which typically develops during the last half of pregnancy. Also with onset in pregnancy ≥20 weeks of gestation, gestational hypertension refers to hypertension without proteinuria. Especially when it occurs remote from term, gestational hypertension frequently progresses to preeclampsia [1]. Preeclampsia and gestational hypertension are important causes of preterm delivery [2,3].

One of the most common and severe complications of preterm delivery is respiratory distress syndrome (RDS). Reports on the relationship between preeclampsia or gestational hypertension and the occurrence of neonatal RDS have been divided, given the difficulty in accounting for confounding variables such as mode of delivery [4–9] and birth weight [4–9]. Neonatal respiratory distress syndrome is caused by insufficient production of surfactant by immature alveolar type 2 pneumocytes. Clinical evidence of RDS appears immediately or within a few hours after birth. It is characterized by tachypnea, retractions, nasal flaring, grunting and cyanosis. Lung maturation is a complicated and incompletely understood process. Vascular endothelial growth factor (VEGF), which binds to the receptors Flk-1 (also known as VEGF receptor-2) and Flt-1 (also known as VEGF receptor-1) is important for pulmonary vascular development [10]. Moreover, VEGF has been shown to increase surfactant protein production [11], and treatment with VEGF prevents fatal respiratory distress in premature mice [12]. Lower VEGF concentrations in tracheal aspirate fluid have been found in preterm infants of women with preeclampsia [13].

Preeclampsia and gestational hypertension are both characterized by an altered angiogenic state with high levels of anti-angiogenic factors, especially when these conditions occur preterm [14–17]. Blood concentrations of sFlt1, a soluble VEGF receptor produced during pregnancy that binds VEGF and placental growth factor (PlGF), increase during the last 2 months of normal pregnancy and attain much greater levels in women with preeclampsia or gestational hypertension [16,18,19]. Studies in both humans and animals suggest that an imbalance in circulating pro- and anti-angiogenic factors resulting in excess blood concentrations of anti-angiogenic factors such as soluble fms-like tyrosine kinase (sFlt1 or sVEGFR1) is responsible for the clinical manifestations of the disease [20–22]. Soluble endoglin, another anti-angiogenic protein, which acts by inhibiting TGF-β signaling, has also been shown to play a pathogenic role in preeclampsia [16,22].

We hypothesized that the high circulating concentrations of anti-angiogenic factors in hypertensive pregnancies would be associated with an increased risk for RDS. We performed a nested case-control study of women who delivered less than 37 weeks of gestation within the Calcium for Preeclampsia Prevention (CPEP) trial. We compared the risk of RDS in infants from normotensive and hypertensive pregnancies and serum angiogenic factor levels among women whose infants did or did not develop RDS.

## Methods

### Participants and specimens

The CPEP trial was a randomized, double-blind clinical trial conducted during 1992-1995 in healthy nulliparous women with singleton pregnancies to evaluate the effects of daily supplementation with calcium on the incidence and severity of preeclampsia [23]. Calcium supplementation had no effect on the incidence, severity, or gestational age at onset of preeclampsia or on the incidence of gestational hypertension.

Enrollment occurred between 13 and 21 weeks of gestation at five medical centers in the United States. Nulliparous women were followed from enrollment until 24 hours after delivery. Women were excluded from study entry if they had a history of hypertension or renal disease, elevated serum creatinine (≥1 mg/ dL), or elevated blood pressure (≥135/85 mm Hg) or proteinuria (≥1+ [30 mg/dL] by dipstick) at either of two screening visits prior to study enrollment at 13–21 weeks of gestation. Serum samples were requested before enrollment, at 26–29 weeks of gestation, 36 weeks of gestation, and when preeclampsia was suspected. Specimens were stored at −70°C.

Among all 4589 women in CPEP, 300 were excluded for the following reasons: 253 lost to follow-up, 21 whose pregnancy terminated before 20 weeks, 17 missing maternal or perinatal outcome data, and 9 with unverified hypertension. Of the remaining 4289, 451 (10.5%) delivered before 37 weeks. Twelve women with gestational proteinuria were excluded. Of the remaining 439 women, 116 with gestational hypertension or preeclampsia were considered the cases, and 323 normotensive women served as controls.

Hypertension was defined as a diastolic blood pressure ≥90 mm on two occasions occurring 4 to 168 hours (1 week) apart. Gestational hypertension was the onset of hypertension after 20 weeks of gestation. Proteinuria was defined by (a) 24-hour urine collection of ≥300mg protein, (b) a single random urine specimen with a protein/creatinine ratio ≥0.35, (c) ≥2+ (100 mg/dL) protein by dipstick in one random specimen or (d) 1+ (30 mg/dL) protein in two random urine specimens occurring 4 to 168 hours apart. Preeclampsia was the occurrence of gestational hypertension and gestational proteinuria within 7 days of each other. Respiratory distress syndrome (RDS) was defined as the acute onset of respiratory distress (grunting, retractions, increased oxygen requirement [FiO2 > 0.4], tachypnea [>60 breaths per minute]) with diagnostic radiographic findings in the absence of evidence for other causes of respiratory distress. Neonatal data were collected prospectively as part of the CPEP trial. Gestational age was determined by the earliest obstetrical ultrasound prior to study enrollment. A small-for-gestational-age infant had a birth weight below the 10th percentile according to U.S. tables of birth weight for gestational age that accounted for race, parity and sex of the infant.[24]

Because specimens could not be linked to identifiable records, the Office of Human Subjects Research of the National Institutes of Health granted the study an exemption from the requirement for review and approval by the institutional review board. Written consent was given by all CPEP study participants prior to enrollment.

### Procedures

Enzyme-linked immunosorbent assays for human sFlt1, soluble endoglin (sEng) and PlGF had previously been conducted in duplicate by R&D Systems Analytical Testing Services (Minneapolis, MN, USA) in all serum specimens obtained from a random sample of 2200 women within the CPEP trial cohort and all other women who developed preeclampsia.[21] Of the 439 preterm mother–infant pairs, 165/323 (51%) normotensive pregnancies and 85/116 (73%) hypertensive pregnancies (66/70 or 94% with preeclampsia and 19/46 or 41% with gestational hypertension) had serum angiogenic factor concentrations measured at least once during pregnancy.

### Statistical analysis

The χ^2^ test was used to compare categorical variables. The *t* test was used to compare continuous variables. For comparison of maternal characteristics, the entire population was used. Comparison of serum angiogenic factor levels was limited to the 57% with samples. Angiogenic factor levels were compared at study enrollment (10–20 weeks) and at midpregnancy (21–32 weeks). When a woman had more than one serum sample within an interval, the sample obtained latest was used. Statistical comparisons of specimens from cases and controls were conducted using linear models, adjusting for gestational age when appropriate. Multivariate logistic regression was used to estimate the odds ratio (OR) of RDS after controlling for gestational age at delivery. Logistic models were developed for RDS. These included the independent variables log sFlt1, gestational age at delivery, birth weight and cesarean section.

## Results

### Characteristics of the women and infants

At the time of enrollment women with preeclampsia or gestational hypertension had higher systolic and diastolic blood pressure, weight and body mass index (BMI) than normotensive women (Table I). No statistically significant differences were noted between hypertensive and normotensive groups in terms of race/ethnicity, parity, maternal age, calcium treatment and gestational age at enrollment. Twice as many normotensive women smoked, but this difference was not statistically significant.

**Table I:** Maternal characteristics at study enrollment for women with delivery <37 weeks in normotensive or hypertensive pregnancies.

	Normotensive(*n* = 323)	Hypertensive(*n* = 116)	*p* value
Age (years)	20.8 ± 4.8*	20.3 ± 3.8	NS
Weight (kg)	65.7 ± 15.4	69.9 ± 19.5	0.04
BMI	25.1 ± 5.5	27 ± 7.7	0.02
SBP (mm Hg)	105.8 ± 8.4	109.2 ± 8.9	<0.001
DBP (mm Hg)	58.9 ± 7.8	63.2 ± 8.1	<0.001
GA at enrollment (days)	122.4 ± 17.6	120.7 ± 17.8	NS
Current smoker	39 (12.1%)	7 (6.0%)	NS
Previous pregnancy	100 (31.0%)	26 (22.4%)	NS
Race/ethnicity			
White, non-Hispanic	83 (25.7%)	26 (22.4%)	
White, Hispanic	50 (15.5%)	15 (12.9%)	NS
Black	184 (57.0%)	70 (60.3%)	
Other, unknown	6 (1.9%)	5 (4.3%)	
Calcium treatment	166 (51.4%)	62 (53.5%)	NS

* Data are presented as mean ± SD or *N* (%). Hypertensive = preeclampsia or gestational hypertension; Calcium treatment = assigned to receive supplemental calcium; BMI, body-mass index; SBP, systolic blood pressure; DBP, diastolic blood pressure; GA, gestational age; NS, not significant.

Perinatal outcomes are presented in Table II. RDS was diagnosed in 87 infants. A significantly greater proportion of preterm infants born to hypertensive mothers had RDS than preterm infants born to normotensive mothers (22.5% vs. 20.9%, *p* = 0.03, after adjustment for gestational age at delivery). The likelihood of developing RDS was greater in infants born of hypertensive pregnancies than of pregnancies complicated by other causes of preterm delivery. After adjustment for gestational age, the odds ratio for the relationship between preeclampsia/gestational hypertension and RDS was 2.18 (95% C.I. 1.08–4.39). As the risk of RDS is mediated in part by other potential confounders, we further examined logistic models. Consistent with previously published studies [8,9], gestational age at delivery and delivery by cesarean section, but not birth weight, were also significantly associated with increased risk of RDS.

**Table II:** Perinatal outcomes for women with delivery <37 weeks in normotensive or hypertensive pregnancies.

	Normotensive(*n* = 323)	Hypertensive(*n* = 116)	*p* value
GA at delivery (weeks)	33.2 ± 4.3*	34.3 ± 3.0	0.002±
Cesarean section	42 (13.0%)	38 (32.8%)	<0.001±
Neonatal death	13 (4.4%)	2 (1.8%)	NS
Birth weight (g)	2080 ± 788	2038 ± 640	NS
SGA	18 (6.1%)	32 (28.3%)	<0.001±
Male	178 (55.1%)	60 (52.2%)	NS
Respiratory distress syndrome	62 (20.9%)	25 (22.5%)	NS±/0.03†
Intraventricular hemorrhage	21 (7.1%)	5 (4.5%)	NS±/NS†
Necrotizing enterocolitis	5 (1.7%)	1 (0.9%)	NS±/NS†

* Data are presented as mean ± SD or *N* (%). Hypertensive = preeclampsia or gestational hypertension; GA, gestational age; SGA, small for gestational age; NS, not significant.

± Indicates significance without adjustment.

† Indicates significance with adjustment for gestational age at delivery.

Hypertensive mothers on average delivered a week later than their normotensive counterparts (34.3 vs. 33.2 weeks, *p* =0.002) and were more likely to have had a cesarean delivery (32.8% vs. 13%, *p* < 0.001). Infants delivered to hypertensive and normotensive mothers had similar birth weights, but infants of hypertensive mothers were more likely to be small for gestational age (SGA). Although the finding was not statistically significant, there was also a slight increase in neonatal mortality in those infants who delivered to normotensive mothers (4.4%) compared with hypertensive mothers (1.8%).

### Angiogenic and anti-angiogenic factor levels

We then compared angiogenic and anti-angiogenic factor levels in normotensive (Table III) and hypertensive pregnancies (Table IV) at baseline (10–20 weeks) and midpregnancy (21–32 weeks) between women who had infants with and without RDS. As expected, regardless of whether pregnancy was normotensive or hypertensive, infants with RDS were born at significantly earlier gestational ages than infants without RDS. Women who remained normotensive during pregnancy delivered fewer infants who developed RDS. When comparing serum pro- and anti-angiogenic factor levels at 10–20 and at 21–32 weeks in normotensive women whose infants did or did not develop RDS, no significant differences were observed (Table III).

**Table III:** Comparison of serum concentrations of sFlt1, PlGF, and sEng among normotensive women who delivered preterm infants with or without respiratory distress syndrome, in specimens collected at 10–20 or 21–32 weeks.

	RDS	No RDS	*p* value
Collected at 10–20 weeks			
Number of women	25	111	
GA at collection (weeks)	17.0 ± 0.5*	16.2 ± 0.2	NS±
GA at delivery (weeks)	29.4 ± 0.8	34.7 ± 0.3	<0.001±
Cesarean section	6 (24.0%)	10 (9.0%)	NS±/NS†
Birth weight (g)	1404 ± 137	2315 ± 55	<0.001±/NS†
SGA	0 (0.0%)	8 (7.2%)	NS±/NS†
sFlt1 (pg/mL)	3895 ± 494	3542 ± 177	NS±/NS†
PlGF (pg/mL)	207 ± 32	146 ± 12	0.05±/NS†
sEng (ng/mL)	6.30 ± 1.0	5.45 ± 0.1	NS±/NS†
Collected at 21–32 weeks			
Number of women	14	117	
GA at collection (weeks)	26.5 ± 0.5	27.1 ± 0.1	NS±
GA at delivery (weeks)	32.1 ± 0.9	35.2 ± 0.2	0.005±
Cesarean section	3 (21.4%)	11 (9.4%)	NS±/NS†
Birth weight (g)	1865 ± 169	2410 ± 42	<0.001±/NS†
SGA	0 (0.0%)	8 (6.8%)	NS±/NS†
sFlt1 (pg/mL)	3645 ± 504	4430 ± 222	NS±/NS†
PlGF (pg/mL)	491 ± 63	706 ± 59	NS±/NS†
sEng (ng/mL)	5.32 ± 0.3	5.67 ± 0.2	NS±/NS†

* Data are presented as mean ± SD or *N* (%). RDS, respiratory distress syndrome; GA, gestational age; SGA, small for gestational age; sFlt1, soluble fms-like tyrosine kinase 1; PlGF, placental growth factor; sEng, soluble endoglin.

± Indicates significance without adjustment.

† Indicates significance with adjustment for gestational age at delivery.

**Table IV:** Comparison of serum concentrations of sFlt1, PlGF and sEng among hypertensive women who delivered preterm infants with or without respiratory distress syndrome, in specimens collected at 10–20 or 21–32 weeks.

	RDS	No RDS	*p* value
Collected at 10–20 weeks			
Number of women	18	55	
GA at collection (weeks)	15.9 ± 0.6*	16.1 ± 0.4	NS±
GA at delivery (weeks)	31.2 ± 0.8	35.4 ± 0.2	<0.001±
Cesarean section	12 (66.7%)	11 (20%)	<0.001±/NS†
Birth weight (g)	1410 ± 155	2207 ± 64	<0.001±/NS†
SGA	8 (44.4%)	17 (30.9%)	NS±/NS†
sFlt1 (pg/mL)	4841 ± 1081	3216 ± 214	0.03±/NS†
PlGF (pg/mL)	108 ± 33	115 ± 14	NS±/NS†
sEng (ng/mL)	8.31 ± 1.9	6.61 ± 0.4	NS±/NS†
Collected at 21–32 weeks			
Number of women	17	53	
GA at collection (weeks)	27.9 ± 0.5	27.6 ± 0.2	NS
GA at delivery (weeks)	31.6 ± 0.8	35.2 ± 0.2	<0.001±
Cesarean section	11 (64.7%)	11 (20.8%)	<0.001±/NS†
Birth weight (g)	1420 ± 156	2201 ± 75	<0.001±/NS†
SGA	9 (52.9%)	15 (28.3%)	NS±/0.045†
sFlt1 (pg/mL)	21516 ± 3734	6998 ± 674	<0.001±/0.01†
PlGF (pg/mL)	215 ± 136	344 ± 56	0.002±/NS†
sEng (ng/mL)	30.63 ± 5.7	16.07 ± 2.6	0.003±/NS†

* Data are presented as mean ± SD or *N* (%). Hypertensive = preeclampsia or gestational hypertension; RDS, respiratory distress syndrome; GA, gestational age; SGA, small for gestational age; sFlt1, soluble fms-like tyrosine kinase 1; PlGF, placental growth factor; sEng, soluble endoglin.

± Indicates significance without adjustment.

† Indicates significance with adjustment for gestational age at delivery.

**Figure 1:**
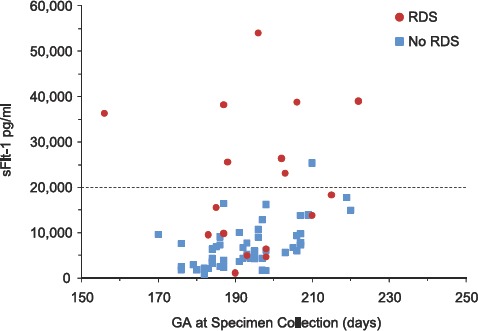
Maternal serum concentrations of sFlt1 (pg/ml) in specimens collected at 21–32 weeks of gestation in women with preeclampsia/gestational hypertension by gestational age at collection (days) and according to whether neonates did or did not develop respiratory distress syndrome (RDS).

In hypertensive pregnancies, women whose infants would develop RDS had substantially higher sFlt1 levels at midpregnancy (Table IV). A scatter plot that contrasts circulating sFlt1 in hypertensive women who delivered infants with and without RDS is displayed in Figure 1. Eight of 17 (47%) sFlt1 values exceeded 20,000 pg/ml at midpregnancy in women with infants who developed RDS, whereas only 1 of 53 (2%) sFlt1 values did so in women whose infants did not develop RDS. This difference in sFlt1 concentrations during midpregnancy (21–32 weeks) remained significant after adjustment for gestational age at delivery (mean 21,516 pg/mL vs. 7000 pg/mL, *p* = 0.01). Logistic modeling indicated that of midpregnancy log sFlt1, gestational age at delivery, birth weight, and cesarean section, only log sFlt1 (pg/ml) (coefficient 1.01, *p* = 0.02) and gestational age at delivery (days) (coefficient−0.06, *p* = 0.002) were significantly associated with the risk of RDS. Controlling for birth weight and cesarean section did not change the association.

Soluble endoglin was elevated, and PlGF was decreased at midpregnancy in hypertensive mothers who delivered infants with RDS, although significance was lost after adjustment for gestational age. Serum concentrations of pro- and anti-angiogenic proteins, after adjustment, were also not significantly different at 10–20 weeks in hypertensive women whose infants would or would not develop RDS (Table IV).

## Discussion

Hypertensive disorders of pregnancy have deleterious effects on both the mother and the fetus. It has been shown in animals and in humans that an excess of circulating anti-angiogenic factors such as sFlt1 and soluble endoglin may play a pathogenic role in the development of maternal proteinuria and elevated blood pressure, the characteristic clinical signs of preeclampsia [17,20–22]. The relationship between preeclampsia and RDS, however, has been a subject of controversy [b4–b9,b25]. In the present study, we report a twofold increased risk of RDS in preterm infants born to nulliparous women with preeclampsia or gestational hypertension, after adjustment for gestational age at delivery.

As preeclampsia [18] and lung immaturity [24] are both characterized by a relative VEGF-deficient state, we tested the hypothesis that angiogenic and anti-angiogenic modulatory factors are further dysregulated in women whose pregnancies are complicated by hypertensive disease and whose infants develop RDS. We found that a higher mean serum sFlt1 level in hypertensive pregnancies is associated with increased risk for RDS. Using logistic models, we also showed that a higher serum sFlt1 concentration in hypertensive pregnancies is associated with increased risk for RDS even after taking into account gestational age at delivery, birth weight, and cesarean delivery. The latter two potential confounders were found to be unrelated to RDS risk when log sFlt1 and gestational age at delivery were included in the model.

Our study confirms the results of investigations by Jelin [8] and Tubman [9] which report that RDS is more common in infants of hypertensive mothers. In our study with relatively small sample size, we detected a *modest* difference in the risk of RDS in preterm infants exposed to maternal preeclampsia or gestational hypertension. Similar to Jelin *et al.*, we also noted that there was no longer a statistically significant increased risk for RDS after controlling for mode of delivery in our study (data not shown), suggesting that the increased risk of RDS may be partially related to the higher frequency of cesarean delivery in women with hypertensive disease. However, when log sFlt1 and gestational age at delivery were included in our logistic model, we found that cesarean delivery as a potential confounder to be unrelated to RDS risk, suggesting a potential correlation between sFlt1 and cesarean delivery. This would reflect the difficulty in disentangling mode of delivery from severity of maternal hypertensive disease as many diseased pregnancies necessitate an iatrogenic operative delivery. In addition to mode of delivery, there may be multiple other confounding factors that could affect our results, notably differences in clinical management, such as the delivery of antenatal steroids, mechanical ventilation and surfactant use.

Information about maternal antenatal steroid use was not available but assumed to be equal among both normotensive and hypertensive women as samples were collected within a limited 3-year period (1992–1995). Before 1995, it was reported that only 20% of premature infants in the United States were treated, with substantial local variations in practice [26]. With the evolution of surfactant and antenatal glucocorticoids, we recognize that there may be effects of improved outcome and that the lack of information about lung maturity induction in this cohort represents a significant weakness for our study. We also noted slightly increased neonatal mortality in the preterm control infants (4.4%) compared with infants who delivered to hypertensive mothers (1.8%); however, this finding did not reach statistical significance, given the sample size. The slight increase in mortality in the preterm control group may be due to many of the same confounding factors as discussed above as well as a lower gestational age.

Hypertensive mothers on average delivered a week later than their normotensive counterparts (34.3 vs. 33.2 weeks). The higher mean gestational age at delivery ≥34 weeks and the increase in RDS risk may reflect a possible clinical practice that antenatal steroids may not have been given to hypertensive women because the pregnancies were greater than 34 ± 0 weeks of gestation. However, our findings would suggest that antenatal steroids are especially critical in pregnancies complicated by gestational hypertension or preeclampsia given the increased RDS risk in these pregnancies. We would argue that the traditional benchmark of 34 weeks of gestation as the cutoff for antenatal steroid delivery be examined and perhaps altered for hypertensive pregnancies.

Our study is also limited by the lack of detailed information regarding the postnatal courses of the preterm infants, especially the severity of RDS and the development of bronchopulmonary dysplasia. A recent study by Hansen *et al.* has shown an association between bronchopulmonary dysplasia and preeclampsia [27]. Also, our study encompasses a broad gestational age range, including all infants delivered at less than 37 weeks. As preterm gestational hypertension has poor pregnancy outcomes [28] and high circulating concentrations of anti-angiogenic proteins [16], we have grouped together gestational hypertension and preeclampsia to obtain adequate power in this study. In studies with larger sample size, there may be not only a higher prevalence of RDS but also a greater disease severity that may be seen in infants with maternal preeclampsia compared with maternal gestational hypertension. As our study was cross-sectional in nature, we cannot exclude the possibility that the rate of rise of sFlt1 during midpregnancy may have had a stronger relationship with subsequent RDS.

The association between hypertensive diseases of pregnancy and RDS is biologically plausible, as both are characterized by relative VEGF deficiency. After comparison of multiple angiogenic factors including soluble endoglin and PlGF, we find a significant association of RDS only with sFlt1. A truncated splice variant of the membrane-bound VEGF receptor Flt1, sFlt1 antagonizes both VEGF and PlGF by binding them in the circulation, preventing interaction with their endogenous receptors [29], and affecting their downstream signaling. Free VEGF and PlGF concentrations in maternal serum are reduced in preeclampsia [20]. Maternal circulating sFlt1 concentration has been shown to correlate with maternal disease severity and to be greater when preeclampsia or gestational hypertension occur preterm [14,17]. There are also increases in anti-angiogenic factor concentrations in the fetal circulation of pregnancies with preeclampsia; however, compared with maternal sera, cord blood levels of sFlt1 are very low [30]. However, previous studies have reported very high concentrations of sFlt1 in third trimester amniotic fluid just prior to delivery. The median sFlt1 concentration in the amniotic fluid of preeclamptic pregnancies (51,040 pg/ml at 33 weeks) was 1.5-fold elevated as compared with the median amniotic fluid sFlt1 concentration of the control group (33,490 pg/ml at 39 weeks) [30]. As circulating sFlt1 in normotensive women rises with increasing gestation, a greater difference in amniotic fluid sFlt1 would be expected if preeclamptic and control specimens had been obtained at similar gestational ages. A recent study also reported elevated sFlt1 in the amniotic fluid of women several months prior to clinical signs of preeclampsia [31]. As the amniotic fluid bathes the developing lung and is an important source of surfactant [32], it is tempting to speculate that the high sFlt1 concentrations in the amniotic fluid of women with preterm gestational hypertension or preeclampsia may inhibit VEGF signaling, leading to surfactant deficiency. Although sFlt1 is secreted into the maternal circulation primarily by the syncytiotrophoblasts [33], the sources of sFlt1 in the fetal circulation and in the amniotic fluid are not known.

In summary, this study supports a modestly increased risk of RDS in neonates delivered from pregnancies with preterm preeclampsia or gestational hypertension. It indicates that RDS in neonates born to these hypertensive pregnancies is correlated with high levels of the anti-angiogenic factor sFlt1 in maternal blood and suggests that inhibition of VEGF by high levels of sFlt1 in amniotic fluid may reduce surfactant. Observational studies in women and experimental studies in animals are needed to examine the relationship between amniotic fluid levels of sFlt1 and the subsequent development of RDS.
